# Tranexamic acid and trauma-induced coagulopathy

**DOI:** 10.1186/s40560-016-0201-0

**Published:** 2017-01-20

**Authors:** Takeshi Nishida, Takahiro Kinoshita, Kazuma Yamakawa

**Affiliations:** Division of Trauma and Surgical Critical Care, Osaka General Medical Center, 3-1-56 Bandai-Higashi, Sumiyoshi-ku, Osaka, 558-8558 Japan

**Keywords:** TXA, CRASH-2 trial, Fibrinolysis, Disseminated intravascular coagulopathy, Transfusion requirements

## Abstract

Tranexamic acid (TXA) is a synthetic derivative of the amino acid lysine that inhibits fibrinolysis by blocking the interaction of plasminogen with the lysine residues of fibrin. Historically, TXA is commonly used for reduction of blood loss in perioperative situations, while recently it has attracted attention for clinical use in the trauma field. In 2010, the Clinical Randomization of an Antifibrinolytic in Significant Hemorrhage 2 (CRASH-2) trial demonstrated that intravenous administration of TXA improved mortality significantly in trauma patients with significant bleeding. After the launch of its sensational results, the main stream treatment protocol in trauma changed worldwide to include TXA administration.

In this review, first we summarize the recent evidence or recommendations in the related guidelines concerning TXA. Also, we next tried to explore in detail not only the benefits but also the harm introduced by TXA in trauma patients, because the main adverse event results for TXA, such as vascular occlusive events in the CRASH-2 trial, are still being discussed in several papers. Thus, we briefly summarized the evidence for the safety of TXA administration by a systematic review method using observational studies. Consequently, the pooled relative risk for venous thromboembolisms was 1.61 (95% CI, 0.86–3.01), indicating a non-significant increase in the venous thromboembolism risk of TXA therapy.

Regarding the basic mechanism, TXA potentially possesses the risk of venous thromboembolisms, so it should be used cautiously and selectively. Further investigation is needed to delineate the optimal targeted trauma patients to earn the maximum survival benefits with minimized risk of thrombotic complications.

## Background

As approximately 1,300,000 individuals die from severe trauma, it is one of the leading causes of death in the world [1]. Hemorrhaging plays an important role in deaths from trauma; it accounts for 30 to 40% of trauma deaths and also increases the mortality of central nervous system injuries [2]. Furthermore, inadequate hemorrhage control in the initial treatment is considered to be the leading cause of potentially preventable deaths occurring after arrival in hospitals [3].

Tranexamic acid (TXA) is a long-established antifibrinolytic drug that was developed in Japan in 1965 [4, 5]. Historically, it is commonly used for a reduction of the blood loss in perioperative situations including cardiac, orthopedic, oral, gynecological, and urological surgeries [6–11]. Several meta-analyses elucidated the efficacy of TXA on the blood transfusion requirements [12, 13]. In 2010, the results of the Clinical Randomization of an Antifibrinolytic in Significant Hemorrhage 2 (CRASH-2) trial, the first multicenter randomized, placebo-controlled trial evaluating the effects of TXA in patients with trauma, were published in *Lancet* [14]. After the launch of its sensational results, the main stream treatment protocol in trauma changed worldwide to include TXA administration [15, 16]. However, unrestricted usage of TXA has been criticized and reconsidered since several studies have pointed out its potential detrimental effects [17–19].

In this review, we will explore the benefits as well as harm introduced by TXA in patients with trauma in order to find out the best treatment option.

### Pathophysiology of trauma-induced coagulopathy

Hemorrhaging can lead to coagulopathy due to multiple factors: shock, acidemia, hypothermia, and hemodilution following resuscitation. A recent study has shown that a hemostatic abnormality is identified in 25% of trauma patients and is associated with increased mortality [20, 21]. The coagulation system in the circulation is activated immediately after trauma by an increased tissue factor production, thrombin generation, and its activation [22]. Simultaneously, tissue hypoxia and ischemia induced by hemorrhagic shock increases the release of the tissue-plasminogen activator (t-PA) from endothelial Weibel-Palade bodies and causes fibrinolysis [23]. Those are the key pathogenesis of the coagulo-fibrinopathy following trauma. In other words, trauma-induced coagulopathy in the early phase of trauma can be categorized into disseminated intravascular coagulation (DIC) with a fibrinolytic phenotype [24, 25]. It leads to systemic bleeding that is not able to be dealt with by surgical procedures and results in a high mortality and morbidity. These findings suggest that treatment against hyperfibrinolysis reduces the mortality of severe trauma with significant hemorrhaging.

An elevation of the plasminogen activator inhibitor-1 (PAI-1) should happen in the coagulation/fibrinolysis system in the following stage. Since it is the principal inhibitor of t-PA, it prevents the formation of plasmin. The gap between the release of t-PA and the increase in PAI-1 in the hypoperfusion status is considered to be several hours [26]. Thus, the phase of the fibrinolytic shutdown follows soon after the DIC phase with the fibrinolytic phenotype. Therefore, antifibrinolytic agents used in the later phase of trauma may not be beneficial and may even be harmful.

### Pharmacological mechanisms of TXA

TXA is a synthetic derivative of the amino acid lysine that inhibits fibrinolysis [27]. Plasma plasminogen is activated and converted to plasmin by t-PA in the presence of fibrin. Plasmin mainly degrades fibrin into fibrin/fibrinogen degradation products. The degradation process requires the connection of the lysine binding sites of plasminogen with the lysine residues on the surface of fibrin. Since TXA has a high affinity for the lysine binding sites of plasminogen, it blocks the interaction of plasminogen with the lysine residues of fibrin and exhibits an antifibrinolytic effect [27].

Because the development of DIC associated with the fibrinolytic phenotype may increase the mortality in trauma, TXA is potentially beneficial to patients who have developed hemostatic abnormalities during the early phase of trauma. On the other hand, a delayed increase in PAI-1 results in the inhibition of fibrinolysis in the later phase [28, 29]. Administration of TXA could accelerate this change and develop detrimental effects when it is used during the fibrinolytic shutdown phase. In fact, numerous basic research studies have demonstrated the pro-thrombotic state enhanced by TXA administration [30–33]. That is, the estimation of the coagulation/fibrinolysis status is quite important to gain the greatest benefit from TXA administration in patients with trauma.

### CRASH-2 trial

The CRASH-2 trial was a large randomized placebo-controlled trial that evaluated the efficacy of TXA in patients with trauma [14]. It included 20,211 patients from 274 hospitals in 40 countries. Adult trauma patients who were within 8 h of injury, with significant hemorrhaging or considered to be at risk of significant hemorrhaging, were eligible for the trial. Significant hemorrhaging was defined as a systolic blood pressure of <90 mmHg or heart rate >110 beats per min, or both. The patients were randomly allocated to receive TXA or a placebo (0.9% saline). TXA was infused 1 g over 10 min as a loading dose, followed by another 1 g over 8 h. The primary outcome was death in hospital within 4 weeks of injury, and the cause of death was categorized into bleeding, vascular occlusions, multiorgan failure, head injury, and others. The secondary outcomes included vascular occlusive events (myocardial infarctions, strokes, pulmonary embolisms (PEs), and deep vein thromboses (DVTs)), receiving blood transfusions, and a transfusion of units of blood products.

The primary outcome data were available for 20,127 patients, 10,060 of whom were allocated to TXA and 10,067 whom were allocated to a placebo. All-cause mortality was significantly lower in the TXA group than placebo group (14.5 vs. 16.0%), and death due to bleeding was also significantly reduced by TXA (4.9 vs. 5.7%). The secondary endpoints including a requirement for surgery, receipt of blood transfusions, and transfusions of units of blood products were equivalent between the two groups. There were no significant differences between the two groups in the occurrence of vascular occlusive events (1.7 vs. 2.0%).

As the expected mechanism of TXA in trauma patients with significant hemorrhaging was the inhibition of fibrinolysis leading to an improved hemostasis, an exploratory analysis that examined the effect of TXA on death due to bleeding according to the time to treatment was published in *Lancet* [34]. Consequently, the risk of death due to bleeding was reduced in two subgroups that received treatment (TXA or placebo) in 1 h or less and between 1 and 3 h from the injury (5.3 vs. 7.7% and 4.8 vs. 6.1%, respectively). On the other hand, TXA increased the risk of death due to bleeding in a subgroup that received treatment more than 3 h after the injury (4.4 vs. 3.1%). It proved that the sooner TXA is infused, the larger the impact it has on death due to bleeding in trauma patients with or at risk of significant hemorrhaging. Moreover, the administration of TXA after 3 h from the injury may be harmful. These results are reasonable because the mechanism of hemostatic abnormalities in trauma is known to change dynamically from DIC with the fibrinolytic phenotype in the early phase into fibrinolytic shutdown with elevated PAI-1 levels in the later phase.

### Cochrane systematic review

A systematic review titled “Antifibrinolytic drugs for acute traumatic injury” was updated in 2015 in the *Cochrane Database Syst Rev*. [35]. Three trials were included in the review, two trials assessed the effect of TXA, and the other assessed that of aprotinin. Since the CRASH-2 trial accounted for more than 99% of the study population, the results from a pooled analysis were predominantly based on the trial. The primary outcome was set as the mortality at the end of the follow-up. Antifibrinolytic drugs reduced the risk of death from any cause (relative risk (RR) 0.90, 95% confidence interval (CI) 0.85 to 0.96). There were no significant differences in the secondary outcomes including surgical intervention, blood transfusions, and the volume of blood transfused. The adverse effects of antifibrinolytic drugs such as PEs, DVTs, myocardial infarctions, and strokes were also evaluated, and it was concluded that there was no evidence that antifibrinolytic drugs had a detrimental effect on the risk of vascular occlusive events.

### Recommendations in the related guidelines

Several guidelines have referred to TXA after the publication of the results of the CRASH-2 trial (Table 1). All of them have recommended an early administration of TXA in trauma patients.

**Table 1:** Recommendations in the related guidelines

Guidelines	Year	Committee	Recommendation
Guidance for diagnosis and treatment of DIC from harmonization of the recommendations from three guidelines.	2013	The Scientific Standardization Committee on DIC of the International Society on Thrombosis Haemostasis	Trauma patients who present with severe bleeding, characterized by a marked hyperfibrinolytic state could be treated with antifibrinolytic agents (moderate quality).
A practical guideline for the hematological management of major haemorrhage.	2015	British Committee for Standards in Haematology	Adult trauma patients with, or at risk of, major hemorrhage, should be given TXA as soon as possible after injury (grade 1A).
The European guideline on management of major bleeding and coagulopathy following trauma: fourth edition.	2016	The pan-European, multidisciplinary Task Force for Advanced Bleeding Care in Trauma	TXA administration was recommended as early as possible to a trauma patient who is bleeding or at risk of significant hemorrhaging (grade 1A)
			Consider administration of the first dose of TXA en route to the hospital (grade 2C)
Major trauma: assessment and initial management.	2016	National Clinical Guideline Centre	Use intravenous TXA as soon as possible in patients with major trauma and active or suspected active bleeding.
			Do not use intravenous TXA more than 3 h after injury in patients with major trauma unless there is evidence of hyperfibrinolysis.

*DIC* disseminated intravascular coagulation, *TXA* tranexamic acid

The guidance for the diagnosis and treatment of DIC by the International Society on Thrombosis and Haemostasis (ISTH) has evaluated that the CRASH-2 trial has provided a moderate quality of evidence [15]. The ISTH guidance recommends the administration of TXA in the early period of management, and to put it concretely, before the levels of PAI-1 and other endogenous antifibrinolytics are elevated. A practical guideline for the hematological management of major hemorrhage by the British Committee for Standards in Haematology also recommends the administration of TXA in adult trauma patients with, or at risk of, major hemorrhaging as soon as possible after an injury (GRADE 1A) [36].

The STOP the Bleeding Campaign established by several societies related to emergency medicine, surgery, anesthesiology, hematology, and intensive care medicine in Europe has published the guidelines on the management of major bleeding and coagulopathy following trauma [16]. It recommends the administration of TXA to trauma patients who are bleeding or at risk of significant hemorrhaging as early as possible (GRADE 1A) and to bleeding trauma patients within 3 h after an injury (GRADE 1B). On the other hand, it recommends that TXA not be given after more than 3 h following an injury. It also refers to the administration of TXA en route to the hospital (GRADE 2C). Similarly, a guideline for the assessment and initial management of major trauma by the National Clinical Guideline Centre recommends the usage of TXA as soon as possible in patients with major trauma and active or suspected active bleeding [37]. It also recommends that TXA should not be infused when more than 3 h has passed after an injury unless there is evidence of hyperfibrinolysis.

### Brief summary

Taken together, all the guidelines above demonstrate the positive recommendation for TXA administration after the CRASH-2 trial in a greater or lesser degree. Now, can we really use TXA for all trauma patients with significant hemorrhaging? Or should we restrict the use of TXA to a limited specific subset of trauma patients? Ian Roberts, one of the authors of the CRASH-2 trial, argued that TXA should be used in all trauma patients at risk of bleeding in a review article in the *J Intensive Care* [38]. Certainly, there is strong evidence that TXA reduces the mortality in bleeding trauma patients, as mentioned above. However, there is still concern about the potential adverse events [14, 18, 39]. We believe that the decision on the utilization of TXA therapy depends on the balance between the efficacy and safety of the therapy.

In the CRASH-2 trial, the rate of vascular occlusive events did not differ significantly between the TXA group and placebo group (TXA 1.7 vs. placebo 2.0%); however, several papers have pointed out the limitations of the results, such as the extremely low rate of venous thromboembolisms (VTEs) reported in the trial [17, 40, 41]. Furthermore, the authors of the CRASH-2 trial admitted that the frequency of vascular occlusive events in the trial could be under-reported [14]. Generally, for the assessment of the safety of the treatment, it is surely acceptable to apply the results of observational studies as well as randomized controlled studies (RCTs). Thus, we next tried to briefly summarize the evidence for the safety of TXA therapy by a systematic review method using both RCTs and observational studies.

### Methods of the systematic review

We conducted a systematic review to evaluate the TXA therapy-related adverse events, especially thrombotic events (VTEs). We searched MEDLINE (source, PubMed) up to July 2016, for articles pertaining to TXA in patients with trauma. We selected clinical trials that met the following characteristics

1. Types of studies: RCTs and observational studies.
2. Types of participants: adult patients following an acute traumatic injury. We excluded studies for only patients with congenital, acquired bleeding disorders, or planned surgical operations.
3. Intervention: intravenous administration of TXA.
4. Control: placebo or no antifibrinolytic drugs
5. Types of outcome measures: VTEs including PEs and DVTs

### Effect of TXA therapy on VTEs

**Fig. 1:**
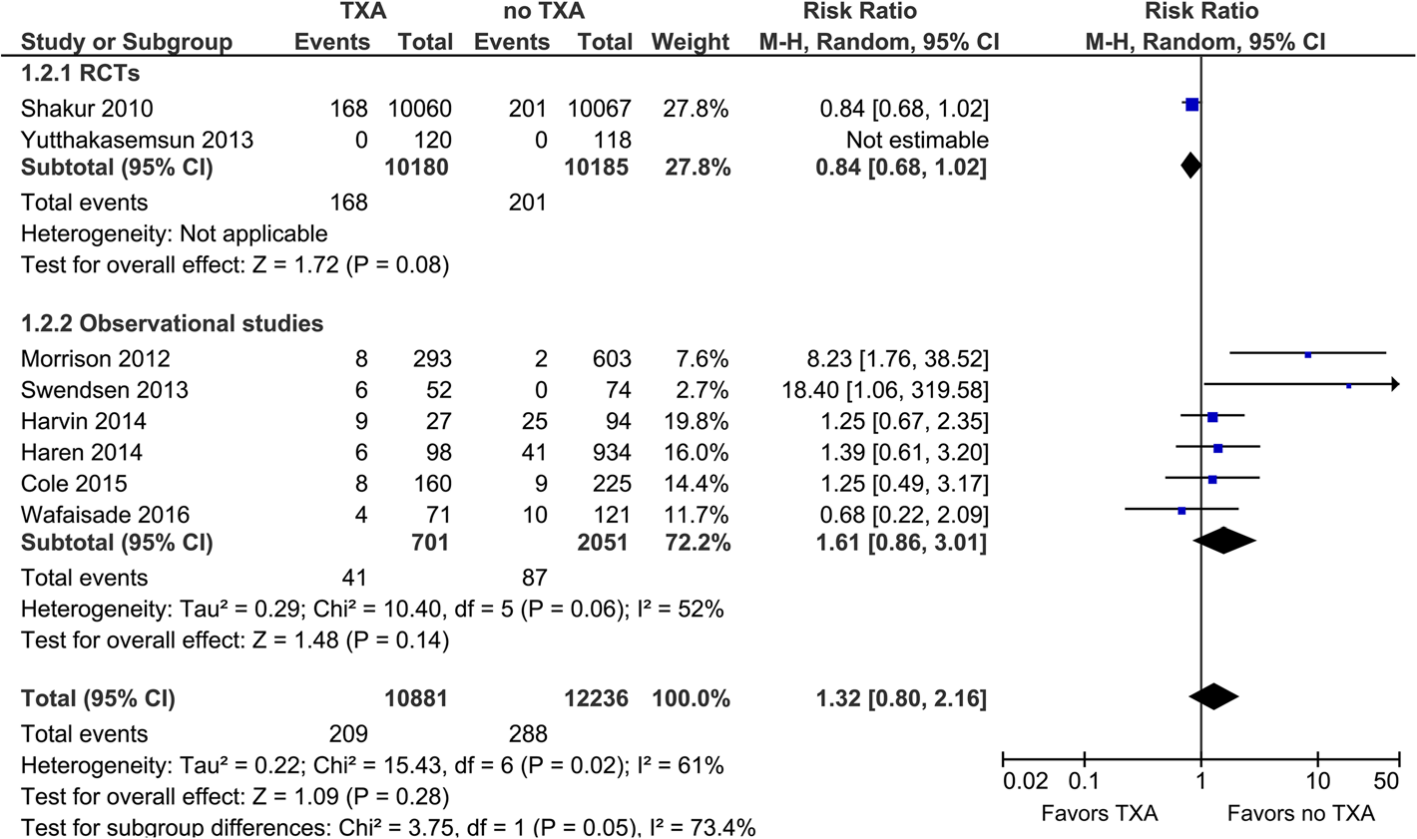
Forest plot of the comparison of tranexamic acid (TXA) versus no TXA for venous thromboembolisms in trauma patients. *RCTs* randomized controlled trials, *M-H* Mantel–Haenszel, *CI* confidence interval

We identified eight studies that evaluated the risk of VTEs related to TXA in trauma patients (20,365 patients/two RCTs and 2752 patients/six observational studies) [14, 19, 38, 41–45] (Table 2). The pooled relative risks for VTEs were 0.84 (95% CI, 0.68–1.02) in the RCTs and 1.61 (95% CI, 0.86–3.01) in the observational studies (Fig. 1). The pooled result of the RCTs was derived from only the CRASH-2 trial. Here, we focused on the results of the observational studies, which indicated a non-significant increase in the VTE risk by TXA therapy. A significant heterogeneity was observed (*I* ^2^ = 52%), and the point estimate of each study varied. Two of the six studies showed a significant increased risk of VTEs by TXA therapy, and three studies showed a non-significant increased risk of VTEs.

**Table 2:** Characteristics of the included studies

Authors	Year	Entry criteria of trauma patients	No. of patients			Mean ISS			Rate of VTE		
			Total	TXA	No TXA	TXA	No TXA	*p* value	TXA (%)	No TXA (%)	*p* value
RCTs											
Shakur et al. [14]	2010	Adult trauma patients with, or at risk of, significant bleeding	20,127	10,060	10,067	N.A.	N.A.	N.A.	1.7^a^	2.0^a^	0.084
Yutthakasemsun et al. [42]	2013	Adult trauma patients with moderate to severe traumatic brain injury (post-resuscitation Glasgow Coma Scale 4 to 12)	238	120	118	N.A.	N.A.	N.A.	0	0	–
Observational studies											
Morrison et al. [38]	2012	Patients who received at least 1 unit of PRBCs within 24 h of admission following combat-related injury	896	293	603	25.2	22.5	<0.001	2.7	0.3	0.001
Swendsen et al. [41]	2013	Adult trauma patients who met triage criteria for serious injury and at least one of the following: hypotension, massive transfusion guideline activation, or transport directly to the operating room or interventional radiology suite	126	52	74	27.1	20.5	0.02	11.5	0	0.004
Haren et al. [43]	2014	Adult trauma patients with hypercoagulable state defined as Greenfield’s risk assessment profile (RAP) ≥10	121	27	94	31	26	0.117	33	27	0.492
Harvin et al. [44]	2014	Adult trauma patients with hyperfibrinolysis determined by rapid thrombelastography	1032	98	934	29	14	<0.001	6.3	4.4	0.389
Cole et al. [19]	2015	Adult trauma patients with severe injury defined as injury severity score (ISS) >15	385	160	225	33	29	<0.05	5	4	ns
Wafaisade et al. [45]	2016	Trauma patients with/without prehospital TXA administration	516	258	258	24	24	0.46	5.6	8.3	0.58

*ISS* injury severity score, *VTE* venous thromboembolism, *RCTs* randomized controlled trials, *TXA* tranexaminc acid, *N.A*. not available, *ns* not significant

^a^These data indicate the rate of pulmonary embolisms

These results suggested that TXA therapy may increase the risk of thrombotic adverse events, but we acknowledged several limitations in this quick review. The risk of bias in the individual studies was serious because of the observational study nature and pooled unadjusted data. Most of the observational studies did not describe the details of the diagnosis protocols or prophylactic treatments for VTEs. Also, a serious imprecision of the pooled estimated risk of VTEs was considered. So, the quality of evidence about the VTE risk of the TXA therapy was very low, and further research is likely to change that estimate.

### Does TXA not increase the VTE rate in the population at high risk of VTEs?

Unlike our quick systematic review shown above, Haren et al. reported that TXA was associated with an improved fibrinolysis but did not increase the VTE rate (TXA 33% vs. no TXA 27%) [43]. The targeted population in this study was ICU trauma patients with a high risk of VTEs defined as a Greenfield’s risk assessment profile of ≥10. In the multivariate logistic regression analysis adjusted for some confounders, TXA was not significantly associated with VTEs. This study was well-designed low risk of bias observational study and easy to understand the main results. Taken together with the conflicting results of our short systematic review, it is difficult to lead to a conclusion as to whether TXA therapy is related to the risk of thrombotic adverse events or not.

## Conclusions

As just described, TXA may have a survival benefit in trauma patients with significant hemorrhaging. However, it is still controversial as to whether or not the administration of TXA is associated with thromboembolic complications. From the perspective of the basic mechanism, TXA potentially possesses the risk of VTEs, so we have to use it cautiously and selectively. Further investigation is needed to delineate the optimal targeted trauma patients to earn the maximum survival benefit with the minimized risk of thrombotic complications.

## Abbreviations

CI: Confidence interval
CRASH-2: Clinical Randomization of an Antifibrinolytic in Significant Hemorrhage-2
DIC: Disseminated intravascular coagulation
DVT: Deep vein thrombosis
ISTH: International Society on Thrombosis and Haemostasis
PAI-1: Plasminogen activator inhibitor-1
PE: Pulmonary embolism
RR: Relative risk
t-PA: Tissue-plasminogen activator
TXA: Tranexamic acid
VTE: Venous thromboembolism
